# Chronic wasting disease associated with prion protein gene (*PRNP*) variation in Norwegian wild reindeer (*Rangifer tarandus*)

**DOI:** 10.1080/19336896.2019.1702446

**Published:** 2019-12-18

**Authors:** Mariella E. Güere, Jørn Våge, Helene Tharaldsen, Sylvie L. Benestad, Turid Vikøren, Knut Madslien, Petter Hopp, Christer M. Rolandsen, Knut H. Røed, Michael A. Tranulis

**Affiliations:** a Department of Basic Sciences and Aquatic Medicine, Faculty of Veterinary Medicine, Norwegian University of Life Sciences, Oslo, Norway; b Norwegian Veterinary Institute, Oslo, Norway; c Norwegian Institute for Nature Research, Trondheim, Norway

**Keywords:** CWD, reindeer, alleles, risk, deer, prions, gene frequency, disease susceptibility, genotype, disease management

## Abstract

The emergence of CWD in Europe in 2016 and the first natural infection in wild reindeer warranted disease management. This led to the testing of 2424 hunted or culled reindeer during 2016–2018, from the infected subpopulation in the Nordfjella mountain range in Southern Norway. To identify any association between *PRNP* variation and CWD susceptibility, we characterized the open reading frame of the *PRNP* gene in 19 CWD positive reindeer and in 101 age category- and sex-matched CWD negative controls. Seven variant positions were identified: 6 single nucleotide variants (SNVs) and a 24 base pair (bp) deletion located between nucleotide position 238 and 272, encoding four instead of five octapeptide repeats. With a single exception, all variant positions but one were predicted to be non-synonymous. The synonymous SNV and the deletion are novel in reindeer. Various combinations of the non-synonymous variant positions resulted in the identification of five *PRNP* alleles (A-E) that structured into 14 genotypes. We identified an increased CWD risk in reindeer carrying two copies of the most common allele, A, coding for serine in position 225 (Ser225) and in those carrying allele A together with the 24 bp deletion.

## Introduction

Chronic wasting disease (CWD) is a transmissible spongiform encephalopathy (TSE) affecting ruminants of the *Cervidae* family [1], like Creutzfeldt-Jakob disease (CJD) in humans, bovine spongiform encephalopathy (BSE) in cattle, and scrapie in small ruminants. CWD expanded its geographic distribution and possibly its prion strain diversity with the emergence in Eurasian reindeer (*Rangifer tarandus*) [2] and moose [3] in Norway in 2016. Previously, this disease had been confined to North America and the Republic of Korea, reported for the first time in Colorado, USA in 1967. Among affected wild cervids in North America are mule deer (*Odocoileus hemionus*), white-tailed deer (*Odocoileus virginianus*), elk (*Cervus elaphus nelsoni*) and moose (*Alces alces*) [1]. Intriguingly, CWD has been identified in North American *Rangifer* only recently and in a captive reindeer [4], despite the potential overlap in cervid habitats. There is a single report of CWD detection in wild-red deer (*Cervus elaphus*) in Europe [5], even though former reports in captive herds [6,7].

CWD is caused by the conversion of the host-encoded cellular prion protein (PrP^C^) into an abnormal isoform (PrP^Sc^, also called prions) [8–10] of which accumulation eventually causes fatal neurodegeneration. Natural transmission and horizontal spread of prions occur in classical scrapie in sheep and CWD. Prions shed to the environment remain infectious for considerable periods [11–13].

Variation in *PRNP* (the gene encoding PrP^C^), particularly within the open reading frame (ORF), is associated with the occurrence of prion disease and may affect prion strain characteristics [14]. Disease susceptibility and progression linked to *PRNP* variation has been reported in elk [15,16], mule deer [17] and white-tailed deer [18,19]. As reported [20–22] 20 known amino acid variant positions within the ORF of *PRNP* are known in cervids including elk, red deer, sika deer (*Cervus nippon*), fallow deer (*Dama dama*), white-tailed deer, mule deer, moose, Chinese water deer (*Hydropotes inermis*) and caribou (*Rangifer tarandus*).

Experiments have shown that reindeer can contract CWD after either inoculation with PrP^Sc^ from white-tailed deer or elk, or by co-housing with infected reindeer. In addition, these experiments demonstrated that *PRNP* genotype probably affects disease susceptibility and progression [23,24].

**Figure 1.**
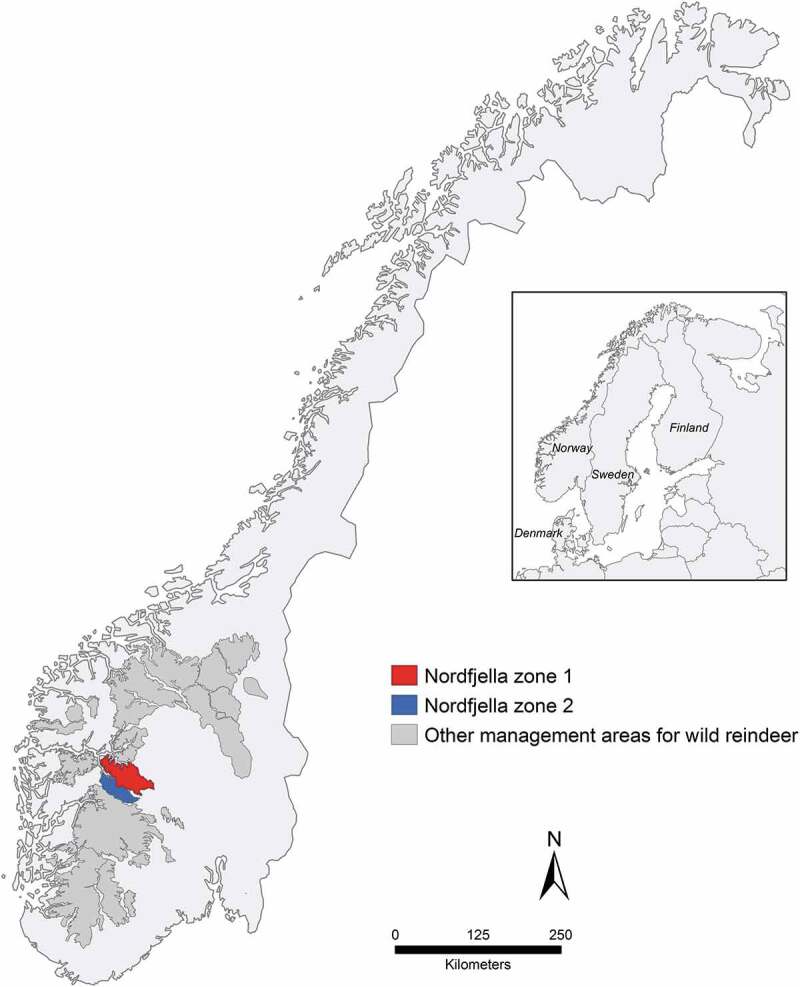
Localization of Nordfjella zones 1, 2 and other wild reindeer management areas in Southern Norway. All cases were detected in zone 1 and sampled in 2016–2018.

The cases of CWD discussed here represent the first known naturally PrP^Sc^ infected reindeer; and all were detected in the Nordfjella mountain area, which is 1 of 23 wild reindeer management areas in Norway (Figure 1). Human infrastructures and reindeer migratory patterns divide Nordfjella into two zones (1 and 2), and the outbreak was limited to zone 1. As a result of the health, economic and biodiversity concerns related to possible spread of CWD from this area [25], the Norwegian government initiated measures to eradicate or at least halt further dispersion of the disease [26], i.e. eradication of the entire subpopulation of reindeer in Nordfjella zone 1 between 2016 and 2018 [27].

We here characterize the coding region of *PRNP* in 120 reindeer, including all 19 CWD cases and 101 controls matched for sex and age categories. The material was analysed for any association between genetic variation and the occurrence of PrP^Sc^. This is the first report of *PRNP* genetic modulation of CWD risk within a reindeer population experiencing an outbreak of the disease. Data presented herein, will be relevant for disease management and allow crude estimation of disease susceptibility at a population-level.

## Results

### PRNP variation in the study population

Sequencing of the ORF of *PRNP* (771 bp) revealed seven variant positions: six single nucleotide variants (SNVs) at positions 4, 6, 385, 505, 526 and 674; and a 24 bp deletion. With the exception of a synonymous substitution at position 6, all variant positions encoded amino acid changes. All variant positions were in Hardy–Weinberg Equilibrium (HWE) (*P*-value = 0.603–1.000). The deletion (249_272del) was located between nucleotide positions 238 and 272 and involved an octapeptide repeat element. Carriers of this deletion tested negative (no PCR product) for the presence of the *PRNP* pseudogene (*PRNPψ*), confirming the presence of the deletion within the functional *PRNP*. The SNV at position 6 and the 24 bp deletion have not been previously reported, other alleles were identical to published *Rangifer tarandus* sequences [21,28]. The sequence data were submitted to GenBank under the following accession numbers: MN784959 (*Rangifer tarandus tarandus* with 6G>A); MN784960 (*Rangifer tarandus tarandus* with 6G>A; 674C>A); MN784961 (*Rangifer tarandus tarandus* with 4G>A; 6G>A; 385G>A; 505G>A); MN784958 (*Rangifer tarandus tarandus* with 249_272del).

The non-synonymous variant sites served as markers to infer *PRNP* alleles encoding unique PrP in the study population. Pairwise analysis of linkage disequilibrium (LD) between 4G>A, 385G>A and 505G>A (D’ = 0.999; r^2^ = 0.999; *P*-value = <0.0001, Supplementary Figure 1), indicated that these positions are genetically linked. LD analyses for SNV at 674 with all other markers gave significantly high D’ and low r^2^ values suggesting that all are linked to 674C. The *PRNP* alleles (Table 1) were named according to amino acid substitution and codon number relative to reference sequence AAZ81474.1, i.e. allele A (Ser225), B (Tyr225), C (deletion), D (Asp176) and E (Met2.Ser129.Met169). Alleles A (Ser225) and B (Tyr225) represented the most common alleles within the study population (Table 1).

**Table 1.** *PRNP* coding sequence alleles and frequencies in Norwegian wild reindeer from Nordfjella zone 1. The allele represents the DNA arrangement within the *PRNP* coding sequence, constructed by phasing non-synonymous variant positions identified in the study population. Variant positions are given at the nucleotide and protein level. Listed positions are characteristic nucleotides and codons for each *PRNP* allele, otherwise identical to reference sequence DQ154293.1 (nucleotide) and AAZ81474.1 (protein).Abbreviations: *PRNP* = prion protein gene; n = number of alleles in the study population.

	*PRNP* open reading frame variant positions						
Nucleotide Protein	4G>AVal2Met	249_272delTrp84_Gly91del	385G>AGly129Ser	505G>AVal169Met	526A>GAsn176Asp	674C>ASer225Tyr	Study population*n* = 240
**Allele nomenclature**							
A	Val	Trp84_Gly91	Gly	Val	Asn	Ser	111 (46.3%)
B	-	-	-	-	-	Tyr	73 (30.4%)
C	-	Trp84_Gly91del	-	-	-	-	23 (9.6%)
D	-	-	-	-	Asp	-	19 (7.9%)
E	Met	-	Ser	Met	-	-	14 (5.8%)

The five *PRNP* alleles (A-E) combined into 14 different genotypes of which A/B (27.5%) and A/A (20.8%) were the most common. Animals homozygous for allele C (deletion) were not observed. Other *PRNP* genotypes were observed at frequencies equal to or below 10%. Detailed descriptions of the variant positions and alleles are given in Supplementary Table 1 and Supplementary Table 2.

Cloning and sequencing of the *PRNP* coding sequence of three heterozygous animals with genotypes A/C and A/E confirmed the identity of the allele sequences separately.

### Expression and Western blot of reindeer PrP

**Figure 2.**
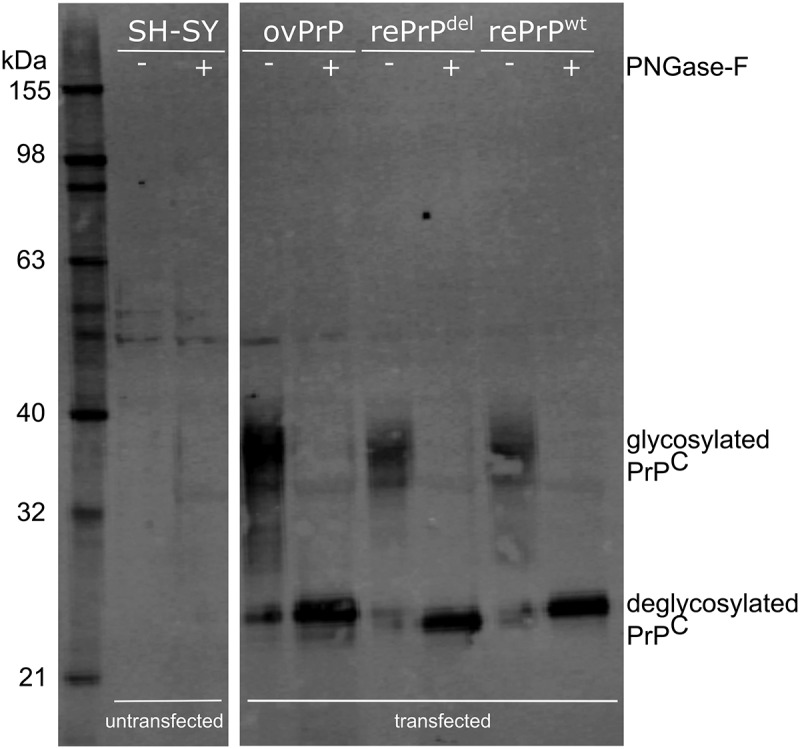
Western blot analysis of PrP^C^ transiently expressed in human neuroblastoma SH-SY5Y cells. Samples were untreated (-) or deglycosylated by PNGase-F treated (+) SH-SY5Y cells, untransfected (SH-SY) and transfected clones with ovine *PRNP* (ovPrP), reindeer *PRNP* with 24 bp deletion (rePrP^del^) and wild type reindeer *PRNP* (rePrP^wt^). Deglycosylated bands from rePrP^del^ and rePrP^wt^ differ with 1 kDa as expected. The membrane was probed with anti-PrP mab P4.

Human neuroblastoma (SH-SY5Y) cells expressing reindeer *PRNP* allele C (deletion; rePrP^del^) and allele A (Ser225; rePrP^wt^) produced similar amounts of PrP^C^ with a similar glycosylation pattern (Figure 2), indicating that PrP^C^ trafficking and post-translational modification with the attachment of N-glycans appears unaffected by the 24 bp deletion.

### PRNP variation in cases and control-groups

**Figure 3.**
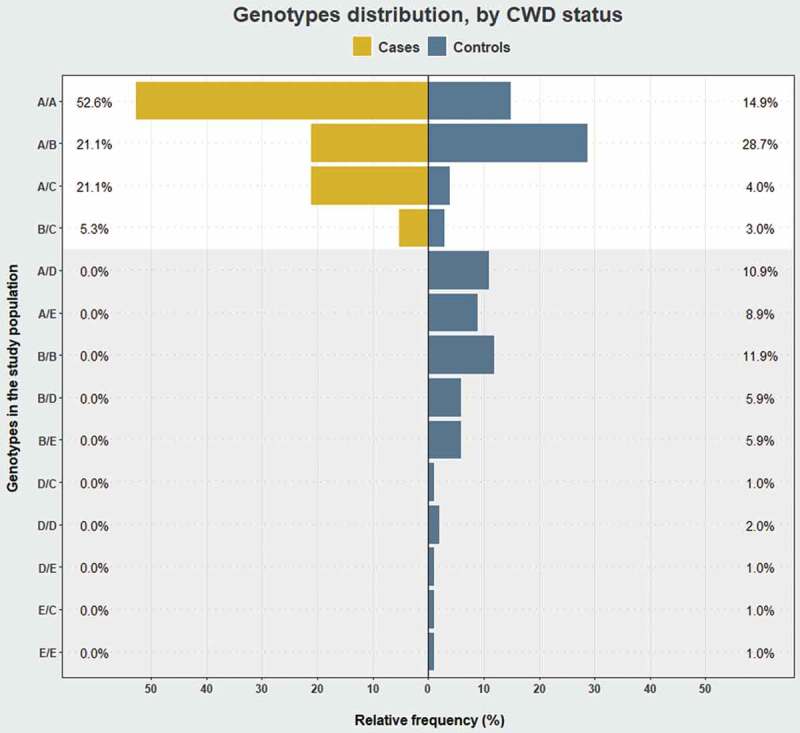
Comparison of *PRNP* genotype frequencies in cases (*n* = 19) and controls (*n* = 101). The relative frequencies between cases and controls were statistically different (*P* < 0.05, Fisher’s exact test). The plot background in white indicates genotypes found in both groups, and in grey, genotypes only found in controls. The B/B genotype was the most frequent genotype exclusive to controls and selected as the baseline for Firth logistic regression with CWD as an outcome and *PRNP* genotype as a predictor.

**Figure 4.**
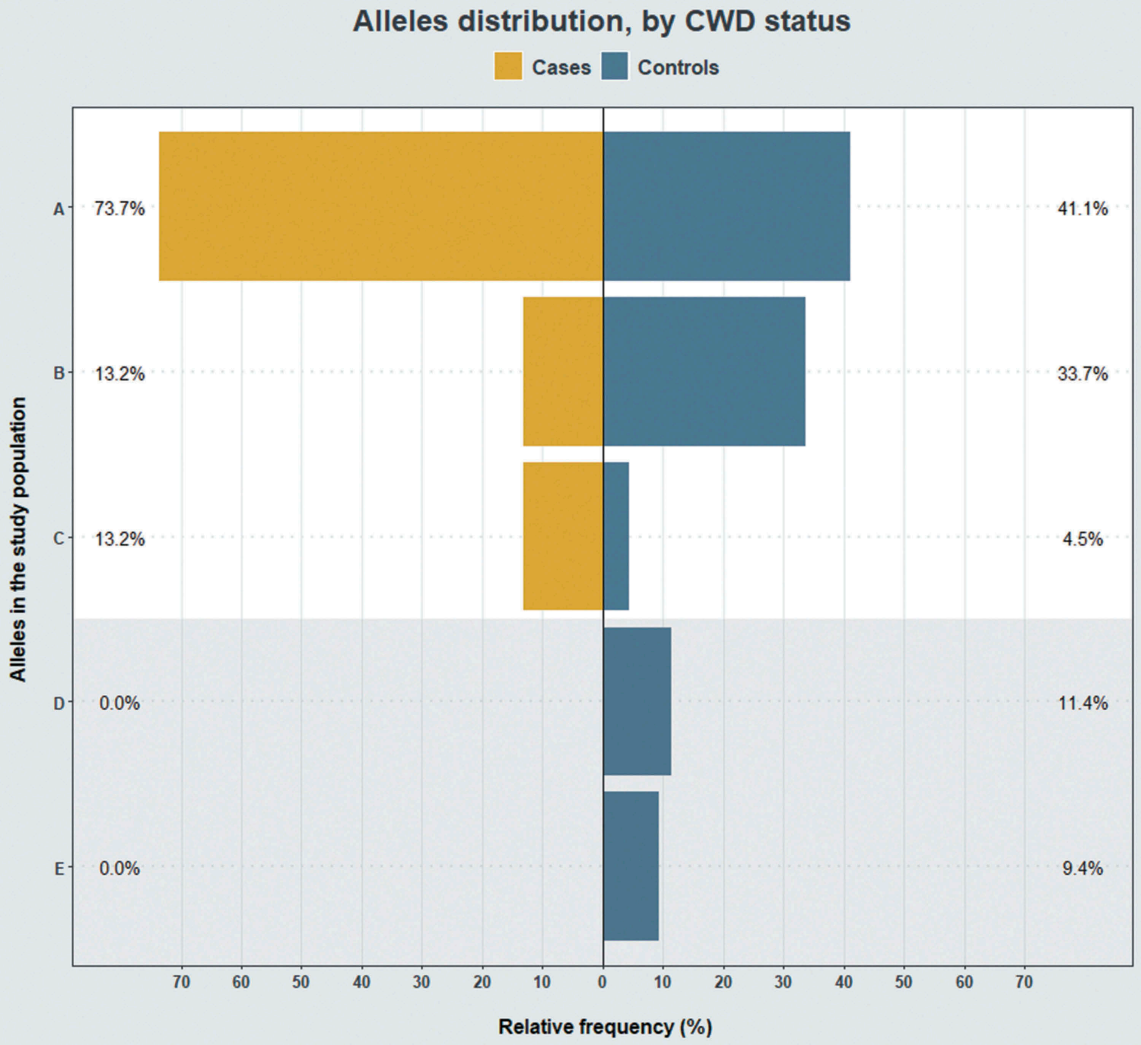
Comparison of *PRNP* alleles’ relative frequency by CWD status in wild reindeer from Nordfjella zone 1, Norway. The relative allele frequencies between CWD cases (*n*= 19) and controls (*n* = 101) were statistically different (*P* < 0.05, Fisher’s exact test). The plot background in white frames genotypes found in cases and controls and in grey, genotypes only found in controls.

The genotype frequencies within the controls were in HWE (*P*-value = 1.000). Only four genotypes were identified among cases, whereas all 14 were represented in the controls (Figure 3). Alleles A (Ser225), B (Tyr225) and C (deletion) were identified in both groups, whereas alleles D (Asp176) and E (Met2.Ser129.Met169) were only detected in controls (Figure 4). *PRNP* allele and genotype frequencies were statistically different between cases and controls (Fisher’s exact test: *P* = 3.59e-05 and 0.01, respectively).

Allele A (Ser225) was the most prevalent in both groups, with a frequency of 73.7% in cases and 41.1% in controls. Similarly, allele C (deletion) was also more prevalent (13.2%) in infected than in non-infected (4.5%). In contrast, allele B (Tyr225) was more frequent in controls (33.7%) compared to cases (13.2%) (Figure 4). Of the common genotypes, A/A at 52.6% was the most prevalent among cases, contrasting with 14.9% in controls amongst which the A/B genotype was the most frequent (28.7%) (Figure 3).

Of the *PRNP* genotypes found exclusively in controls (Figure 3), B/B was the most frequent and served as the baseline genotype for the regression analysis. Firth logistic regression analysis suggested an increase in CWD risk on the shift from B/B to either A/A or A/C (Supplementary Table 3). Given the significance of genotypes A/A and A/C as predictors of CWD, we performed a similar regression analysis to test the effect of copy number of these alleles and CWD status. The model revealed that carrying two copies of allele A (Ser225) and/or allele C (deletion) is a risk factor for CWD, significantly increasing the odds ratio (O.R.) (O.R: 42.39; 95% C.I. = 5.12–5534.03) (Table 2).

**Table 2.** Analysis of association between numbers of carried copies of either allele A (Ser225) or allele C (deletion) and CWD risk in wild reindeer from Nordfjella zone 1. Regression coefficients and associated statistics from Firth logistic regression. Asterisk indicates a significant value. Abbreviations: S.E. = standard error; C.I. = confidence interval.

Predictor	Regression coefficient	S.E.	95% C.I.		*P*-value	Odds ratio
Constant	−4.04	1.452	−8.89	−2.08	<0.001	-
No copies	Baseline					
Single copy of allele A or C	1.75	1.52	−0.48	6.64	0.144	5.75
Two copies of allele A or C	3.75	1.494	1.63	8.62	<0.001*	42.39

## Discussion

This is the first study to analyse *PRNP* coding variation among cases and controls in a population of wild reindeer sampled during an outbreak of CWD. Our data show that of the five alleles detected (A-E), A (Ser225) and C (deletion) were overrepresented among the cases and that all affected reindeer carried allele A, allele C or both. Allele C differs only from allele A in the octapeptide deletion. By comparing the expression of alleles A (rePrP^wt^) and C (rePrP^del^) in transfected cells, we showed as expected, that loss of a single octarepeat does not affect translation of *PRNP* mRNA or further post-translational protein modification with the attachment of N-glycans. Both alleles A (Ser225) and C (deletion) were overrepresented among the cases when compared to the controls, almost twice and treble, respectively. Given their overrepresentation in the CWD-affected reindeer, we combined the two alleles when performing the Firth logistic regression analysis, which returned a high odds ratio, indicating an increased CWD risk.

The allele B (Tyr225) carrying the Ser225Tyr substitution was more frequent among controls compared to cases. An analogous substitution (Ser225Phe) in wild mule deer results in a longer CWD incubation time in heterozygotes [29]. This clinical onset likely relates to the structural effect Ser225Phe has on PrP, that is proposed delay of PrP^C^-to-PrP^Sc^ conversion [30], a feature observed *in vitro* for Ser225Tyr [31].

Alleles D (Asp176) and E (Met2.Ser129.Met169) were not detected among cases but constituted 20.8% of all alleles identified in controls. In sheep, a similar substitution at codon 176 (Asn176Lys) is considered protective against classical scrapie challenge [32,33]. Likewise, reindeer carrying a single copy of the allele E (Met2.Ser129.Met169) did not develop CWD [23] after oral challenge. In Moore et al., reindeer carrying allele E had longer survival-times following intracranial exposure [24]. In the same experiment, a reindeer with a genotype carrier of E, found dead without showing clinical signs ~13 months post-intracranial inoculation, had no histopathological lesions or PrP^Sc^ deposition at post-mortem examination.

The different distribution of the alleles by CWD status is in accordance with *in vivo* and *in vitro* data comparing the allele A (Ser225) with other reindeer *PRNP* alleles. The index case reported by Benestad, Mitchell [2] was of genotype A/A, i.e. an amino acid sequence identical to that found in reindeer susceptible to CWD by oral exposure [23]. Our allele A (Ser225) corresponds to isolate QGAQ of Haley, Rielinger [31] in a Real-Time Quaking-Induced Conversion assay. In that study, the amplification abilities of isolate QGAQ across alleles QGAQ, 225Y (similar to allele B) and SSM (similar to allele E) showed that 225Y and SSM had significant lower amplification rates than QGAQ. These observations are consistent with a scenario in which alleles B (Tyr225) and E (Met2.Ser129.Met169) are less likely to form the initial PrP^Sc^ than allele A (Ser225).

Our data support the notion that *PRNP* genetic variation modulates CWD susceptibility rather than conferring complete resistance. This is in agreement with experimental observations of reindeer-developing CWD after intracranial inoculation regardless of *PRNP* genotype [24].

The 3D structure of the C-terminal globular domain of PrP is well conserved in phylogenetically distant organisms and consists of three α-helices of which α2-helix (α2) and α3-helix (α3) are linked by a stabilizing disulphide bond. Moreover, a short anti-parallel β-sheet (β1 and β2 strands) is present, with a loop-structure connecting β2 and the α2 [34]. Interestingly, the structure of the β2-α2 loop, encompassing residues 165–175 (codons 168–178 in reindeer *PRNP*), has been shown to be important for interspecies transmission of prion disease in mice models [35] and the rigidity of the β2-α2 loop, common to cervids [36], at least partly explains the efficient transmission of CWD between cervid species [37]. Recent data suggest that long-range stabilizing interactions between the β2-α2 loop and α3 affect prion propagation [38,39]. For instance, 3D simulations have shown that Ser225Phe, results in a rearrangement allowing a stabilizing hydrogen bond to be established between the β2-α2 loop and α3 that could explain the reduced susceptibility associated with this substitution [30]. A similar effect could be caused by Ser225Tyr in reindeer allele B (Ser225), which could explain the rarity of this allele among the CWD cases.

The identity of the alleles was verified by direct DNA sequencing following cloning and by their occurrence in homozygous animals. With the exception of the novel synonymous substitution (6G>A) and the deletion (249_272del), all *PRNP* alleles were identical to published *Rangifer tarandus* sequences [21,28]. These observations collectively confirm the *PRNP* allele identities as described in our study population.

Comparison of our data with former studies in *Rangifer* spp. show that Gln226 is found constitutively within the genus. Different residues at codon 226 appear to influence the selection and propagation of CWD strains as observed in transgenic [40] and gene-targeted [39] mice bioassays. Mice Gln226 (GtQ226^+/+^) and Glu226 (GtE226^+/+^) respond differently to challenge with the same CWD prion inocula, and the derived prions appear to have distinct conformational properties. Hypothetically, this could denote that diseased deer (Gln226) and elk (Glu226) propagate prions with different strain properties [39]. As appearing in reindeer, Gln226 could select strains that more likely resemble properties of CWD prions in diseased deer (Gln226) rather than in diseased elk (Glu226), which can be important for the risk of interspecies CWD transmission. Demographic factors such as dispersal and population structure impact *PRNP* variation [41]. While the substitutions Ser138Asn (413G>A) [23,28,42] and Asn146 = (438C>T) [28,42] are observed in North American *Rangifer*, but not in the Nordfjella population, the constitutive Ser138 and substitutions at codons 176 and 225 are in accordance with *PRNP* variation previously identified in Norwegian reindeer [21] and absent in North America. The variation patterns in *PRNP* from *Rangifer* spp. as described in different geographic locations encourages further study of their relationship with CWD susceptibility.

The detrimental effects of CWD are progressively being unveiled in North America [43] which implies that the establishment of endemic CWD in Europe could be devastating over time. Further, the culled Nordfjella subpopulation constituted approximately 10% of the remaining wild European tundra reindeer population. Efforts to repopulate Nordfjella zone 1, therefore, have been politically warranted. Any restocking has to rely on a strategy that is realistic when it comes to preventing reinfection [25].

The description of *PRNP* variation in a naturally infected reindeer population, here presented in association with CWD risk, provides insight into the most susceptible *PRNP* genotypes. The current knowledge does not support genetic resistance towards CWD in cervids. However, knowledge of modulated risk could act as a management tool in both wild and semi-domesticated populations.

## Materials and methods

### Source population

The Nordfjella wild reindeer management area is located in central Southern Norway (Figure 1). It is divided into zones 1 and 2, which are approximately 2000 km^2^ and 1000 km^2^ respectively. The two areas are separated primarily by a road, and the presence of tourist cabins and hiking trails also leads to low connectivity between these subpopulations. The initial CWD cases recorded in 2016 were confined to zone 1; thus, the entire subpopulation was culled in a process completed by April 2018 [44].

During 2016–2018, 2424 reindeer were removed from Nordfjella zone 1, which represented our source population. All culled animals were examined for CWD at the Norwegian Veterinary Institute (NVI), which is the national TSE reference laboratory and a World Organization for Animal Health (OIE) CWD reference laboratory. The *medulla oblongata* and retropharyngeal lymph node tissues were tested as a pooled sample for initial screening for the presence of PrP^Sc^ [45]. Pooled samples testing ELISA positive (TeSeE ® SAP ELISA, Bio-Rad) were then re-tested using individual tissues and further verified by Western blot (TeSeE ® Western, Bio-Rad). Any reindeer found PrP^Sc^ positive in at least one tissue (brain or lymph node) was classified as a case. Subsequently, all samples were stored frozen (−20⁰C).

### Study population

A matched case–control study was performed to identify associations between *PRNP* genotypes and CWD risk. The study design included a minimum of four controls (PrP^Sc^ negative) per case (PrP^Sc^ positive) with a match on age category and sex. The rationale being the low prevalence of CWD, and that age and sex could act as confounding variables in the genetic association analysis.

For all cases and controls, the sex and the age categories were based on autopsy (cases) or information from the hunters (controls). The study population included all CWD cases with matched controls in the source population.

We analysed *PRNP* variation in a total of 120 wild reindeer: 19 cases and 101 controls. The cases were sampled between March 2016 and April 2018. The controls were randomly selected from a selection pool consisting of stored samples from Nordfjella zone 1 collected between March 2016 and October 2017. The matching on age category and sex for cases and controls was close to equal (Table 3).

**Table 3.** Distribution of sex and age category in wild reindeer from Nordfjella zone 1, Norway by CWD status (cases and controls) is presented as absolute and relative frequencies. Young = 15–22 months old < adult.

Sex	Age category	Cases (*n*= 19)	Controls (*n* = 101)
Male	Young	1 (5.3%)	2 (2.0%)
	Adult	12 (63.2%)	68 (67.3%)
Female	Young	0 (0.0%)	3 (3.0%)
	Adult	6 (31.6%)	28 (27.7%)

### PRNP sequencing

Genomic DNA was extracted from brain tissues with DNeasy® Blood and Tissue kit (Qiagen, Oslo, Norway). The ORF of *PRNP* was amplified using PCR primers Ce19_F (5ʹ-ATTTTGCAGATAAGTCATC-3ʹ) and Ce778_R (5ʹ-AGAAGATAATGAAAACAGGAAG-3ʹ) designed by O’Rourke, Spraker [46]. The PCR reaction contained 2 µl genomic DNA as template, 2 µl dNTPs mix (4 x 2.5 mM) (WVR, Radnor, PA, USA), 0.6µl forward and reverse primers (10 pmol) (Eurofins genomics, Luxembourg, Luxembourg), 2 µl Key Buffer (15 mM MgCl_2_) (VWR, Radnor, PA, USA), 0.1 µl Taq DNA polymerase (5 U/μl) (VWR Radnor, PA, USA) and purified water to a final reaction volume of 20 µl. The PCR amplification started with an initial cycle at 95°C for 2 min, followed by 36 cycles at 95°C for 30 s, 51°C for 30 s and 72°C for 45 s, then a final cycle at 72°C for 10 min.

PCR products were visualized in a 1.5% agarose gel and purified by illustra™ ExoProStar™ 1-Step (GE Healthcare, Uppsala, Sweden). The sequencing reaction was performed using the initial PCR primers and a BigDye™ Terminator v3.1 Cycle Sequencing Kit (Applied Biosystems, Foster City, CA, USA) according to the manufacturer recommendations. Finally, sequence data for both strands were generated using a 3500xL Genetic Analyser (Applied Biosystems, Foster City, CA, USA).

Some samples required an additional PCR sequencing reaction for optimal visualization of the initial sequence in the ORF. For this purpose, we used primer set Ce19_F and Prp-157_R (5ʹ-ACTTCCCTGTCCCGGGTAT-3ʹ, in-house). A group of samples were additionally tested for the presence of the *PRNP* pseudogene (*PRNP*ψ), with primers 369/224 [46] with visualization of the PCR product on a 1.5% agarose gel utilizing PCR parameters as given above.

Sequences were aligned with SeqScape v3.0 (Applied Biosystems) and edited in MEGA7 version 7.0.26 [47]. The Human Genome Variation Society nomenclature guidelines (version 15.11) were used to describe the sequence variants [48] and the nomenclature was checked with Mutalyzer 2.0.28 [49]. The description of *PRNP* variant positions and alleles is based on reference sequences DQ154293.1 for nucleotides and AAZ81474.1 for amino acids. Sequences were treated as unphased genotypes to infer two alleles per individual by a conservative best-fit approach, which considered the minimum possible number of alleles together with pairwise Linkage disequilibrium (LD) analysis between variant positions.

### Cloning

*PRNP* coding sequences were amplified (standard conditions) from the genomic DNA of three heterozygous animals with PCR primers modified to introduce *EcoRI* and *NotI* restriction sites at the ends of the amplicon (Ce19m_F: 5ʹ-AGTCGAATTCATTTTGCAGATAAGTCATC-3ʹ and Ce778m_R: 5ʹ-TGACGCGGCCGCAGAAGATAATGAAAACAGGAAG-3ʹ). The amplicons were then cloned into the pCI-neo Mammalian Expression Vector (Promega, Madison, WI, USA) using standard cloning techniques. Selected clones were sequenced as described above to independently confirm each allele.

### Cell culture and transfection

The human neuroblastoma cell line SH-SY5Y (RRID:CVCL_0019) (Sigma-Aldrich, St. Louis, MO, USA) was cultured as previously described [50].

Plasmid constructs encoding either allele A (Ser225) (SH-SY5Y rePrP^wt^) or allele C (deletion) (SH-SY5Y rePrP^del^) were transiently transfected into the SH-SY5Y cells using jetPRIME® (Polyplus-transfection® SA, Illkirch, France) following the manufacturer’s protocol.

### Western blot

Cells were lysed with homogenizing buffer (Tris HCl 50 uM, NaCl 150 mM, EDTA 1 mM, DOC 0.25%, NP40 1%) supplemented with cOmplete™ protease inhibitor cocktail (Roche, Basel, Switzerland). The protein concentration was determined with the Protein Assay kit I (Bio-Rad, Hercules, CA, USA). For each sample, 20 µg protein was deglycosylated with PNGase-F (New England BioLabs Inc) in accordance with the manufacturer’s guidelines, and 20 µg protein were analysed untreated. The samples were denatured using sodium dodecyl sulphate (SDS) loading buffer (Invitrogen) and Sample Reducing Agent (Thermo Fisher Scientific) before separation by polyacrylamide gel electrophoresis (PAGE) on a 12% Criterion™ XT Bis-Tris gel (Bio-Rad). Proteins were transferred to a polyvinylidene difluoride (PVDF) membrane (GE Healthcare, Chicago, IL, USA) and the membrane blocked with 5% non-fat dried milk in TBS – Tween. The membrane was then incubated with primary antibody (P4 mouse anti PrPC; Ridascreen Biopharm AG, Darmstadt, Germany) 1:1000, and secondary antibody (anti-mouse IgG conjugated to alkaline phosphatase; Invitrogen) 1:1000. Detection with EFC™ substrate (GE Healthcare) was performed on a Typhoon 9200 imager (Amersham Biosciences, Sunnyvale, CA, USA).

### Statistical analysis

The statistical analyses were executed using R version 3.5.2 [51] and RStudio version 1.1.456 [52] using *genetics* and *logistf* packages. Results were plotted using *dplyr* and *ggplot2* packages.

Categorical variables were summarized into absolute (counts) and relative (percentages) frequencies for cases and controls. Contingency tables were analysed by Fisher’s exact test (or Chi-square test if suitable). Tests were two-sided and a *P*-values <0.05 were interpreted as significant.

HWE fitness was tested in the control group for genotypes at each variant position within the *PRNP* sequence; a *P*-value <0.05 was interpreted as a departure from HWE. Linkage disequilibrium between variant position pairs was measured by D’ and r^2^. After interpretation of the *PRNP* alleles, phased genotypes of the *PRNP* sequence were evaluated for HWE fitness in the controls. *PRNP* allele and genotype associations with CWD status were tested by Fisher’s exact test.

The Firth method reduces small-sample bias in the logistic model due to the small number of cases. Thus, Firth logistic regression was used to investigate the relationship between CWD outcome and *PRNP* genotype. The CWD risk was assessed in terms of the odds ratio, which resulted from the exponentiation of the regression coefficients.
