# Supplementary material for: Chronic wasting disease associated with prion protein gene (PRNP) variation in Norwegian wild reindeer (Rangifer tarandus)
Source: Prion. 2019 Dec 18;14(1):1–10. doi: 10.1080/19336896.2019.1702446 (PMC6959294; doi:10.1080/19336896.2019.1702446)
Supplement: Supplemental Material [file kprn-14-01-1702446-s001.docx]

Taylor & Francis Word Template for journal articles-

Supplementary data

Supplementary table 1. Variant positions in *PRNP* coding sequence from wild reindeer from Nordfjella zone 1. Variant descriptions are in accordance to the HGVS nomenclature definition, using the three-letter amino acid symbol. DNA and protein level descriptions are reported in comparison to reference sequences DQ154293.1 (nucleotide) and AAZ81474.1 (protein). Frequency analysis is based on variance at the nucleotide level.

Abbreviations: *PRNP*= prion protein gene; HGVS = Human Genome Variant Society; DNA = deoxyribonucleic acid.

| DNA variation | Predicted protein variation | Minor Allele Frequency |
| --- | --- | --- |
| 4G>A | Val2Met | 0.08 |
| 6G>A | Val2= | 0.02 |
| 249_272del | Trp84_Gly91del | 0.06 |
| 385G>A | Gly129Ser | 0.08 |
| 505G>A | Val169Met | 0.08 |
| 526A>G | Asn176Asp | 0.10 |
| 674C>A | Ser225Tyr | 0.30 |

Supplementary table 2. *PRNP* alleles in wild reindeer from Nordfjella zone 1. The alleles represent the DNA arrangement within *PRNP* coding sequence predicted to encode unique prion proteins. Alleles were constructed by phasing non-synonymous variant positions from the study population. Variant descriptions are in accordance to the HGVS nomenclature definition, using the three-letter amino acid symbol. DNA and protein level descriptions are reported in comparison to reference sequences DQ154293.1 (nucleotide) and AAZ81474.1 (protein).

Abbreviations: *PRNP*= prion protein gene; HGVS = Human Genome Variant Society; DNA = deoxyribonucleic acid.

| Allele | DNA variant | Predicted protein variant |
| --- | --- | --- |
| A | DQ154293.1 | AAZ81474.1 |
| B | DQ154293.1:c.[674C>A] | AAZ81474.1:p.[(Ser225Tyr)] |
| C | DQ154293.1:c.[249_272del] | AAZ81474.1:p.[(Trp84_Gly91del)] |
| D | DQ154293.1:c.[526A>G] | AAZ81474.1:p.[(Asn176Asp)] |
| E | DQ154293.1:c.[4G>A;385G>A;505G>A] | AAZ81474.1:p.[(Val2Met;Gly129Ser;Val169Met)] |
| A2 | DQ154293.1:c.[6G>A] | AAZ81474.1:p.[(Val2=)] |

Supplementary table 3. Analysis of association between *PRNP* genotypes and CWD risk in wild reindeer from Nordfjella zone 1 culled between March 2016 and April 2018. Regression coefficients and associated statistics from Firth logistic regression. B/B genotype served as the baseline for being the most frequent genotype restricted to the controls. Asterisk indicates a significant value.

Abbreviations: *PRNP* = prion protein gene; CWD = chronic wasting disease; S.E. = standard error; C.I. = confidence interval.

| Predictor | Regression coefficient | S.E. | 95% CI | | *P*-value | Odds ratio |
| --- | --- | --- | --- | --- | --- | --- |
| Constant | -3.219 | 1.501 | -8.072 | -1.193 | 0 | - |
| B/B | Baseline |  |  |  |  |  |
| A/A | 2.829 | 1.555 | 0.613 | 7.722 | 0.008* | 16.94 |
| A/B | 1.339 | 1.587 | -1.015 | 6.253 | 0.308 | 3.81 |
| A/C | 3.219 | 1.659 | 0.715 | 8.182 | 0.009* | 25.00 |
| A/D | 0.083 | 2.128 | -5.175 | 5.342 | 0.967 | 1.09 |
| A/E | 0.274 | 2.143 | -4.988 | 5.537 | 0.893 | 1.32 |
| B/C | 2.372 | 1.856 | -0.764 | 7.449 | 0.137 | 10.71 |
| B/D | 0.654 | 2.183 | -4.62 | 5.929 | 0.752 | 1.92 |
| B/E | 0.654 | 2.183 | -4.62 | 5.929 | 0.752 | 1.92 |
| D/C | 2.12 | 2.754 | -3.266 | 7.543 | 0.354 | 8.33 |
| D/D | 1.609 | 2.419 | -3.72 | 6.952 | 0.463 | 5.00 |
| D/E | 2.12 | 2.754 | -3.266 | 7.543 | 0.354 | 8.33 |
| E/C | 2.12 | 2.754 | -3.266 | 7.543 | 0.354 | 8.33 |
| E/E | 2.12 | 2.754 | -3.266 | 7.543 | 0.354 | 8.33 |


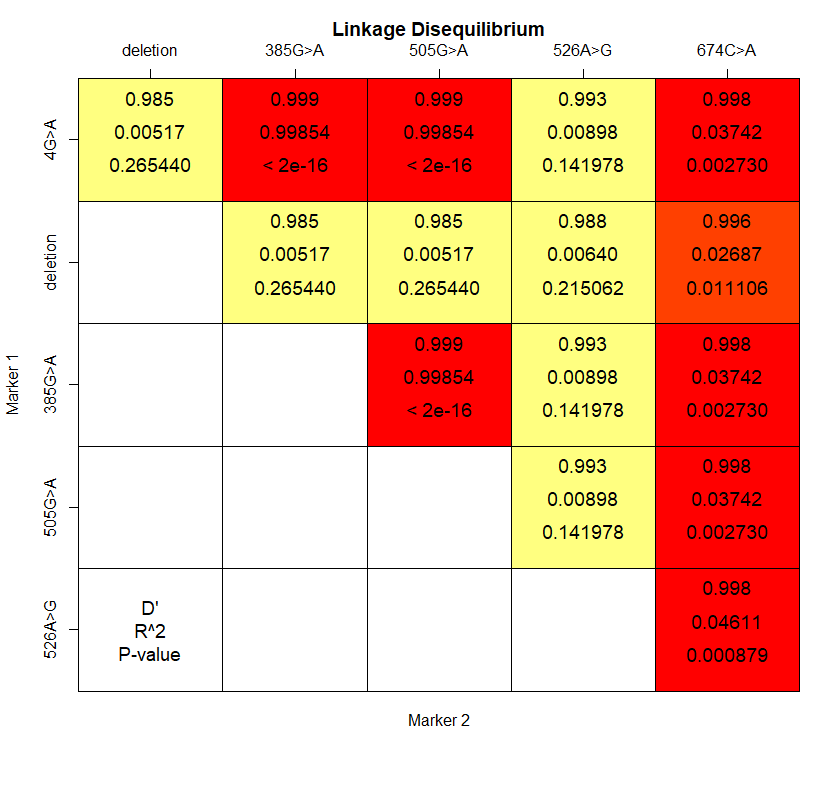


Supplementary figure 1. Pairwise LD analysis between non-synonymous variant positions (nucleotide) in *PRNP* coding sequence. Each frame summarizes LD between marker 1 and marker 2 measured by D’, R^2 and *P*-value (order shown in the lower left frame). Color-shading based on *P*-value where red indicates a high significance.

Abbreviations: LD = linkage disequilibrium; *PRNP* = prion protein gene; D’ = scaled disequilibrium statistic; R^2 = mean squared correlation coefficient
